# The Impact of Changes in Paraspinal Muscle Tissue on Lumbar Percutaneous Intradiscal Radiofrequency Therapy

**DOI:** 10.3390/jcm13226696

**Published:** 2024-11-07

**Authors:** Emel Güler, Tuğçe Yavuz Mollavelioğlu, Nalan Çelebi

**Affiliations:** 1 Division of Pain Management, Department of Anesthesiology and Reanimation, Faculty of Medicine, Gazi University, Ankara 06560, Turkey; 2 Division of Pain Management, Department of Anesthesiology and Reanimation, Faculty of Medicine, Hacettepe University, Ankara 06230, Turkey; tugceyavuz@hotmail.com.tr (T.Y.M.); nalanmd@hotmail.com (N.Ç.)

**Keywords:** lumbar degenerative disc, Goutallier classification, pain, Oswestry Disability Index

## Abstract

**Background/Objectives:** This study aimed to assess whether fatty changes in paraspinal muscle tissue negatively affect pain relief and functional outcomes, measured by the Oswestry Disability Index (ODI), in patients undergoing intradiscal bipolar radiofrequency thermocoagulation for lumbar degenerative disc (LDD) disease. Fatty changes in paraspinal muscles, often associated with sarcopenia, are known to negatively influence treatment outcomes. However, there is limited research on how these changes affect pain and functional capacity following intradiscal procedures. **Methods:** In this study, data from 59 patients treated for LDD were analyzed. Pain severity was measured using the Numerical Rating Scale (NRS), and the ODI was recorded before the procedure and at 1, 3, and 6 months post-procedure. Fatty changes in the paraspinal muscle tissue were evaluated using the Goutallier classification based on pre-procedure T2-weighted MRI scans, while disc degeneration was assessed using the Pfirrmann classification. **Results:** The results showed significant positive correlations between the Goutallier and Pfirrmann classifications and pain levels at all time points post-procedure (*p* < 0.05). Notably, the relationship between NRS scores and Goutallier classification was stronger than that with Pfirrmann classification (*p* < 0.05). ODI scores were also significantly correlated with both Goutallier and Pfirrmann classifications at each time point, with a stronger association observed between ODI and Goutallier classification than with NRS (*p* < 0.05). **Conclusions:** These findings suggest that fatty changes in the paraspinal muscle tissue may significantly influence treatment outcomes and should be considered during pre-treatment evaluations. Further research is needed to explore this relationship more comprehensively.

## 1. Introduction

Chronic lower back pain (LBP) is caused by various etiological factors that can create a serious economic burden and negatively affect daily life. Lumbar degenerative discs (LDD) cause chronic lower back pain, and many factors are considered in their pathophysiology. The formation of granulation tissue during the healing of fissures in the annulus fibrosus, an increase in cytokine release, and the growth of nociceptive nerve fibers in this area are among the reasons for pain [1]. Degeneration is observed at different severities and can be graded using a classification system developed by Pfirrmann et al. using magnetic resonance imaging [2].

In the diagnosis of lumbar degenerative disc disease (LDD), clinical symptoms and radiological findings are evaluated together. Chronic low back pain, especially pain exacerbated by sitting and bending, is the most common symptom of LDD [1]. The most important imaging modality used for diagnosis is magnetic resonance imaging (MRI), with the Pfirrmann classification being applied to assess the severity of disc degeneration [2]. This classification grades the degenerative state of discs based on criteria such as disc structure homogeneity, signal intensity, and disc height [2]. Additionally, procedures like discography, which involve the injection of contrast material into the disc to provoke pain, can help confirm discogenic pain [3]. Moreover, degenerative changes such as fat infiltration or muscle loss in the paraspinal muscles, particularly in elderly patients, are often associated with LDD and can exacerbate the severity of the disease [4,5,6].

As a result, segmental stabilization of the lumbar vertebral segment deteriorates, leading to discogenic pain [1,3]. Another factor that causes disruption in segmental stabilization is changes that occur in the paraspinal muscle tissue. Studies have shown that changes in muscle mass resulting from aging, an increase in fat tissue, and alterations in muscles contribute to back pain, and are also effective in the process of disc degeneration [4,5,6]. The severity of intra-muscular fat changes can be evaluated and graded using magnetic resonance imaging (MRI), which is called the Goutallier classification [7]. Sarcopenia has emerged because an increase in adiposity negatively affects the prognosis and treatment success of many musculoskeletal disorders. Sarcopenia, first defined in 1989, can be described as both a loss of muscle mass and loss of muscle function in the process of physiological aging [8,9]. However, a previous study provided a specific definition for spine-related sarcopenia. Regardless of its definition, the detrimental effects of sarcopenia on back problems have been highlighted [10].

In addition to conservative treatments, various interventional procedures have been applied for the treatment of LDD. One of these is intradiscal bipolar radiofrequency thermocoagulation. The link between LDD and paraspinal muscle changes, and how these affect therapeutic approaches like radiofrequency thermocoagulation, could be explored in more depth. The mechanism of action involves generating heat using electrothermal cannulas placed into the disc to coagulate neural and inflammatory tissues and reduce collagen fibrils, thereby reducing nociceptive input [11]. However, despite using the same treatment techniques, the response to treatment varies among patients, emphasizing the importance of evaluating surrounding anatomical structures. Although the study’s objective is clearly stated, it lacks a formal hypothesis regarding how these changes in paraspinal muscle tissue might affect the success of this treatment modality. Clarifying a hypothesis would strengthen the framework of the study. For example, it could predict that patients with higher degrees of intramuscular fat or sarcopenia might exhibit poorer outcomes following thermocoagulation due to decreased segmental stability and increased fat infiltration, as has been observed in other studies involving interventional treatments for degenerative lumbar conditions [12,13].

Studies on patients who have undergone back surgery due to different pathologies have shown that those with low muscle mass exhibit a lower quality of life and slower recovery, among other effects [14,15,16,17]. Additionally, more attention should be given to surrounding anatomical structures, as they play a crucial role in patient outcomes after radiofrequency treatments. The purpose of this study was to evaluate the effects of intradiscal bipolar radiofrequency thermocoagulation treatment in patients diagnosed with LDD on the fatty changes in paraspinal muscles at the level of the treated disc using current imaging methods, and to assess its impact on pain and quality of life after the procedure. Given the critical role of paraspinal muscles in spinal stability, we hypothesized that increased fat infiltration into these muscles might result in less effective outcomes following intradiscal procedures. Understanding this relationship is essential for optimizing treatment approaches in LDD patients. Performing the pre-treatment evaluation of paraspinal muscle morphology, using techniques such as the Goutallier classification, could be essential for determining which patients are likely to benefit most from this intervention [17,18].

## 2. Materials and Methods

In this retrospective study, the records of patients who applied to the Hacettepe University Faculty of Medicine, Department of Pain Management, between 1 January 2021 and 30 February 2023 and received intradiscal bipolar radiofrequency thermocoagulation treatment after being diagnosed with LDD were included in the examination. Patient age, height, weight, and BMI were calculated and recorded. Consent forms were prepared for the accessible patient group and consent was obtained.

The inclusion criteria for the study were patients who applied to the Algology Polyclinic with a diagnosis of LDD and had available lumbar MRI images. Discography, which has been proven as a reliable provocative test in identifying discogenic pain, was performed by injecting contrast material into the disc to ensure precise targeting of the affected disc level for thermocoagulation. This test, combined with clinical evaluations, allowed for the accurate selection of the disc level for treatment [19,20]. Patients included were those aged between 18 and 85 years who showed more than a 50% increase in pain in response to intradiscal injection of 2 mL contrast material given as discography and underwent the procedure after the evaluation of their MRI images, and who agreed to the evaluation of their existing images. Patients with immobility or severe physical disability that could limit mobility were excluded from the study to ensure that such conditions would not confound the outcomes. Patients aged <18 or >85 years were excluded from the study. Patients who did not agree to the evaluation of their existing images were excluded from this study.

Evaluation scales to be used during patient records were the following:

The Oswestry Disability Index (ODI) was used to determine functional improvement. It consists of ten items measuring the intensity of pain, personal care, lifting, walking, sitting, standing, social life, sleep, travel, and pain levels. Each item was rated from 0 to 5 [21].

Radiological evaluation: The Goutallier classification was used to assess the extent of fat infiltration into the paraspinal muscles. The Pfirrmann classification was used to determine the severity of the disc degeneration.

**Figure 1:**
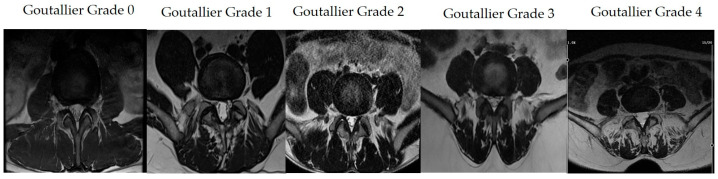
Goutallier classification on MRI images.

Goutallier classification:

Grade 0: No intra-muscular fat.
Grade 1: Very little fat present.
Grade 2: Fat is less than muscle.
Grade 3: Fat and muscle ratio is equal.
Grade 4: Fat exceeds muscle (Figure 1) [22].

Pfirrmann classification:

Grade 1: The structure of the disc is homogeneous, with bright hyperintense white signal intensity and normal disc height.
Grade 2: The structure of the disc is not homogeneous, with a hyperintense white signal. The distinction between the nucleus and annulus is clear, and the disc height is normal regardless of the presence of horizontal gray bands.
Grade 3: the structure of the disc was not homogeneous, with moderate gray signal intensity. The distinction between the nucleus and annulus is unclear and the disc height is normal or slightly decreased.
Grade 4: The structure of the disc is not homogeneous, with a hypointense dark-gray signal intensity. The distinction between the nucleus and annulus fades and the disc height is normal or moderately decreased.
Grade 5: The structure of the disc is not homogeneous, with a hypointense black signal intensity. The distinction between the nucleus and annulus fades, and the disc area collapses [23].

**Figure 2:**
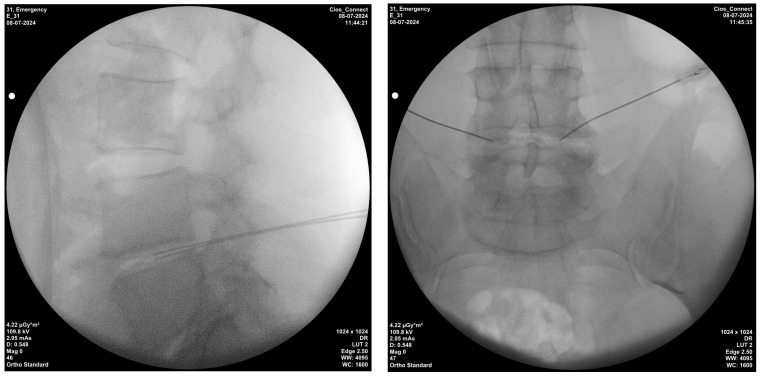
Techniques of intradiscal bipolar radiofrequency thermocoagulation injection; lateral and posterior–anterior fluoroscopic views.

Bipolar radiofrequency thermocoagulation was performed in a sterile room. Sedation was administered to all monitored patients beforehand. Patients placed in the prone position had their treatment area cleaned and covered with a sterile drape, the disc level and entry point where the interventional procedure was performed were localized using C-arm fluoroscopy with the tunnel vision technique. Appropriate disc level and needle placement were confirmed with a posteroanterior fluoroscopic view and advanced, whereas depth was confirmed with a lateral fluoroscopic view (Figure 2). After the procedure, a mixture of 1 mL physiological saline solution and an antibiotic (cephalosporin) was injected into the disc, and the procedure was completed. A 20-gauge, 15 cm needle, and 10 mm active-tip catheter were inserted into the disc. Following a lack of response (no sensory or motor response) to 2 Hz and 50 Hz at 2 V, a lesion was consecutively created at 50 °C for 2 min, 55 °C for 2 min, 60 °C for 2 min, and 65 °C for 4 min using an NT1100 RF generator (NeuroTherm, Middleton, MA, USA) [19].

This study was approved by the Hacettepe University Clinical Research Ethics Committee (decision no. SBA 23/021). All the procedures were performed in accordance with the ethical rules and principles of the Declaration of Helsinki.

### Statistical Analysis

The SPSS version 25 statistical package program was used for the statistical analyses. Descriptive statistical methods (mean, median, frequency, percentage, minimum, and maximum) were used to summarize the data. The Shapiro–Wilk test was used to test the normality of continuous variables. The Wilcoxon signed-rank test was used to investigate differences between the two groups for continuous variables that did not show a normal distribution. The choice of non-parametric tests, such as the Wilcoxon signed-rank test, was based on the Shapiro–Wilk test results, which indicated that the continuous variables did not follow a normal distribution. Transformations to achieve normality were considered; however, due to the nature of the data and the relatively small sample size, transformations were not expected to sufficiently normalize the data. Therefore, non-parametric tests were preferred to preserve the validity of the results. The significance level for all tests was set at *p* < 0.05. A total of 59 patients were included in this study. The power of the test for the data was found to be 93.65% using the GPower 3.1 software program. This power calculation was based on the effect size observed in previous studies examining similar interventions and outcomes in LDD patients. A detailed power analysis was conducted before the study to determine the necessary sample size to detect clinically significant differences with a power of at least 80%. The observed power of 93.65% reflects the strength of the study’s findings given the sample size and effect size. Due to continuous variables not following a normal distribution and the dependence of the sample, the non-parametric Wilcoxon signed-rank test was used for the calculations.

## 3. Results

A total of 59 patients, 32 of whom were female and 27 were male, were included in the study. The age of the patients ranged from 33 to 82 years, with an average of 56.47 ± 12.59. The duration of pain was 23.98 ± 14.38 months. The demographic and clinical data of the patients are shown in Table 1.

Table 2 presents the correlation coefficients and significance test results for the values of the Goutallier and Pfirrmann classifications with the NRS and ODI variables before the procedure and at 1, 3, and 6 months. Although the relationships between the Goutallier and Pfirrmann classifications and NRS status before the procedure were insignificant, they were significant and positively correlated with the values at 1, 3, and 6 months. According to the magnitude of the relationship with the Pfirrmann classification, NRS scores at 1, 3, and 6 months were more strongly positively correlated with the Goutallier classification. Therefore, the Goutallier classification was considered more successful.

The ODI values were significantly and positively correlated with the Goutallier and Pfirrmann classifications before the procedure and at 1, 3, and 6 months. It can be seen that the ODI values have a stronger relationship with the Goutallier classification than the NRS values.

Table 3 presents the average age, pain duration, NRS, and ODI according to the Goutallier classification of the patients, as well as the results of the Kruskal–Wallis test. In the p-values obtained, there were differences in the averages among the Goutallier groups for variables other than the pre-procedure NRS (*p* < 0.05).

Patients with a Goutallier classification of 0 had a lower average age than the others, and those with a classification of 1 were younger than those with classifications of 2, 3, and 4. The patients with the lowest amount of pain had a Goutallier classification score of 0. Patients with a Goutallier classification of 3 and 4 had significantly higher mean NRS scores at 1, 3, and 4 months than those with a Goutallier classification of 0, 1, and 2. It is also understood that the ODI mean before and at 1, 3, and 6 months for patients with a Goutallier classification of 0 were lower than those in groups 1, 2, and 3, while the averages for those in group 4 were higher.

The mean, median, minimum–maximum, and standard deviation for the NRS and ODI variables of the patients are presented in Table 4.

Table 5 presents the differences between the averages of NRS and ODI variables obtained from patients before the procedure and at 1, 3, and 6 months after the procedure as well as the *p*-values of the Wilcoxon signed-rank tests used to test the significance of these differences. The results showed that the mean NRS and ODI significantly decreased from before the procedure to 3 months, with no significant difference between 3 and 6 months (*p* < 0.05).

The average ODI was higher before the procedure than at the other time points; however, there were no significant differences between the averages obtained at 1 month and beyond.

## 4. Discussion

In this study, in patients who underwent intradiscal bipolar radiofrequency thermocoagulation therapy with a diagnosis of LDD, the reduction in ODI scores at the 6-month follow-up was analyzed in relation to the Goutallier and Pfirrmann classifications, which served as the primary outcome measures. The pre-procedure findings showed a significant and positive correlation with the values recorded at 1, 3, and 6 months post-procedure, particularly when compared with the NRS scores. As for NRS, it was found that the decrease in NRS scores at 1, 3, and 6 months post-procedure was significantly and positively correlated. As a secondary outcome, the average NRS and ODI scores significantly decreased from the pre-procedure state to 3 months, with no statistically significant difference between 3 and 6 months. A possible explanation for this plateau effect could be that intradiscal bipolar radiofrequency thermocoagulation initially reduces pain through controlling inflammatory processes and promoting nerve tissue healing. However, as muscle degeneration and fat infiltration persist, these benefits may plateau around the 3-month mark, with the reduction in pain becoming less pronounced by the 6-month follow-up. Upon reviewing the literature, it was concluded that the relationship between paraspinal fatty changes and intradiscal bipolar radiofrequency thermocoagulation therapy for LDD treatment was evaluated for the first time in this study.

The concept of sarcopenia has gained great importance with the increase in the elderly population. Its diagnosis is made by evaluating several parameters such as a decrease in muscle mass and the concept of fragility it brings along. Fragility is a geriatric syndrome that accompanies functional loss in many systems and is associated with fatty changes within the muscle that negatively affect both muscle function and strength [24]. The relationship between this change and chronic low back pain has been demonstrated, and the paradox of whether fatty changes cause low back pain or vice versa has been investigated [25].

Various interventional procedures are performed to treat the causes of chronic low back pain, and the response to treatment varies. The importance of evaluating patients from multiple perspectives has emerged as the answer to this question. In a study that included 183 patients who underwent percutaneous epidural adhesiolysis treatment with a diagnosis of LDD, paraspinal intramuscular fatty changes were evaluated at the lumbar 3–4 vertebra level, and patients were divided into two groups: those aged > 65 years and those aged < 65 years. In female patients aged > 65 years, fatty changes negatively affected the success of the procedure, whereas in male patients aged > 65 years and in both sexes aged < 65 years, fatty changes were unaffected. Fatty changes were recorded at a single level of the lumbar region. Disc degeneration was not evaluated using any classification, and functional capacity was not assessed [26]. In our study, the evaluation of fatty changes based on the Goutallier classification showed that an increased fat percentage had a negative effect on both the functional capacity and pain score. The changes in fat content were assessed based on the specific level at which the procedure was performed. An increase in disc degeneration negatively affects the decrease in pain intensity and increase in functional capacity after the procedure. However, its relationship with age has not yet been evaluated in geriatric populations.

In a retrospective study conducted by Kim et al., lumbar epidural steroid injections were administered to 245 patients aged ≥ 65 years. Poor analgesic results on pain assessment after the procedure are associated with advanced age, opioid use, foraminal stenosis severity, and excess fat changes in the paraspinal muscles. The severity of fat changes was classified as grades 0, 1, or 2. Based on these results, changes in fat content should be considered independent risk factor [27]. In another study involving 275 patients who received cervical interlaminar epidural steroid injections, fat changes in the multifidus muscle at the C5-6 level were evaluated in relation to treatment responses. It has also been suggested that fat changes should be considered as an independent risk factor for treatment success. Furthermore, disc degeneration was evaluated, but was not associated with the success of injection therapy [28]. In our study, we found that both intramuscular fat changes and an increase in disc degeneration negatively affected pain and functional capacity, which were used to evaluate procedure success.

The treatment protocol we used, the intradiscal bipolar radiofrequency thermocoagulation method, can be applied at different temperatures. Li et al. included 23 patients with discogenic low back pain in their study, and the procedure was performed at 85 °C for 180 s. A statistically significant decrease in pain, ODI, and analgesic consumption was found in patients followed for one year [12]. In our study, a statistically significant decrease in pain and ODI was observed after 6 months compared to baseline, but no significant differences were found at the 3rd and 6th month assessments. The average age of patients in the study was 43.0, while in our study it was 56.47, and additional environmental and psychosocial factors that we did not evaluate may have contributed to this result.

Zeng et al. applied intradiscal monopolar treatment to 84 patients and intradiscal bipolar radiofrequency thermocoagulation treatment to 84 patients under computerized tomography guidance. The procedures were performed at 50, 60, and 70 °C for 60 s each, 80 °C for 90 s, and 92 °C for 100 s. Patient follow-ups were conducted on days 7, 30, and 180 using the Macnab criteria and a visual analog scale. A statistically significant improvement in pain score was found in patients treated with bipolar therapy; however, the efficacy scores were high for both procedures, with no significant difference between them [29]. In our study, both the decreases in pain intensity and disability scores were significant. However, there were differences in the thermal procedures applied, follow-up parameters, and duration between our study and this study.

### Strengths and Limitations

This study has several limitations. First, the retrospective nature of our study and the absence of a control group had some limitations. Second, although fat changes were evaluated as follow-up parameters, other parameters used in the diagnosis and follow-up of mobilization and sarcopenia were not used, which is another limitation. Another limitation is that the evaluation of fat changes made with preprocedural imaging was not re-evaluated during post-procedural follow-up. The absence of post-procedural MRI may affect the accuracy of the Goutallier classifications over time and limits the ability to observe changes that may occur during the follow-up period. Another limitation of the study was that no additional rehabilitation program was recommended to the patients. They were advised to continue their daily living activities as before the procedure, but no further recommendations were provided.

## 5. Conclusions

In conclusion, before deciding on an intradiscal procedure, it is essential to evaluate all relevant parameters. Assessing paraspinal muscle morphology, particularly the degree of fat infiltration, should be regarded as a critical prognostic factor when setting therapeutic goals and selecting candidates for intradiscal bipolar radiofrequency thermocoagulation. Identifying patients with greater muscle degeneration can help tailor treatment plans more effectively and improve overall outcomes. Therefore, paraspinal muscle assessment has emerged as an important evaluation criterion for clinicians when making procedural decisions. However, this study also highlights the need for clinicians to consider patient selection more carefully in the context of intradiscal radiofrequency therapy. Specifically, the degree of paraspinal muscle degeneration and fat infiltration should be considered when determining which patients might benefit most from this treatment. This could lead to more personalized and effective treatment plans in clinical decision making.

Future research should focus on validating these findings through more rigorous study designs, such as prospective cohort studies or randomized controlled trials. These studies would provide clearer evidence on how factors like muscle degeneration and fat infiltration affect long-term outcomes and treatment efficacy. By conducting these studies, a clearer path forward for improving patient selection criteria can be established.

## Figures and Tables

**Table 1:** Demographic and clinical information of the patients.

Gender	BMI	Surgical Status	Procedure Level	Goutallier Classification	Pfirrmann Classification
Female 32 (54.2%)	Underweight 1 (1.7%)	Non-surgical 53 (89.8%)	L3-4 5 (8.5%)	Grade 0 11(18.64%)	Score 1 0
Male 27 (45.8%)	Normal weight 13 (22.0%)	Surgical 6 (10.2%)	L4-5 34 (57.6%)	Grade 1 17 (28.81%)	Score 2 8 (13.56%)
	Overweight 26 (44.1%)		L5-S1 20 (33.9%)	Grade 2 16 (27.12%)	Score 3 33(55.93%)
	Obese 18 (30.5%)			Grade 3 11 (18.64%)	Score 4 18 (30.51%)
	Morbidly obese 1 (1.7%)			Grade 4 4 (6.78%)	Score 5 0

BMI: body mass index.

**Table 2:** Correlation coefficients and significance test results for the values of the Goutallier and Pfirrmann classifications with the NRS and ODI variables before the procedure and at 1, 3, and 6 months.

Spearman’s Rho Analysis	Goutallier Classification	*p* Value	Pfirrmann Classification	*p* Value
**NRS pre-procedural**	−0.112	0.300	0.066	0.567
**NRS 1st month**	0.380	0.000	0.277	0.014
**NRS 3rd month**	0.322	0.022	0.261	0.018
**NRS 6th month**	0.352	0.001	0.262	0.018
**ODI pre-procedural**	0.394	0.000	0.263	0.013
**ODI 1st month**	0.432	0.000	0.237	0.025
**ODI 3rd month**	0.449	0.000	0.381	0.008
**ODI 6th month**	0.461	0.000	0.269	0.011

NRS: Numerical Rating Scale; ODI: Oswestry Disability Index; *p* < 0.05.

**Table 3:** Averages of age, pain duration, NRS, and ODI according to Goutallier classification and results of the Kruskal–Wallis Test.

Goutallier Classification						
	0	1	2	3	4	*p* Value
**Age**	45.45	51.41	60.88	63.45	71.50	0.001
**Duration of pain**	13.45	31.94	24.38	22.00	23.00	0.014
**NRS pre-procedural**	7.64	7.47	7.63	6.73	7.75	0.324
**NRS 1st month**	2.55	3.06	3.63	4.55	5.50	0.010
**NRS 3rd month**	2.36	2.47	3.25	4.45	5.00	0.028
**NRS 6th month**	2.36	2.35	3.38	4.45	5.50	0.008
**ODI pre-** **procedural**	38.18	43.18	52.00	50.00	63.00	0.002
**ODI 1st month**	14.73	20.59	27.50	38.55	48.50	0.002
**ODI 3rd month**	14.00	16.94	25.75	38.55	47.50	0.001
**ODI 6th month**	14.36	15.65	25.13	39.09	48.00	0.001

NRS: Numerical Rating Scale, ODI: Oswestry Disability Index, *p* < 0.05.

**Table 4:** Mean, median, minimum–maximum, and standard deviation of continuous variables for the patients.

NRS	Median (Min–Max)	Mean ± SD	ODI	Median (Min–Max)	Mean ± SD
**NRS pre-procedural**	7.00 (5.00–10.00)	7.42 ± 1.16	ODI pre-procedural	46.00 (30.00–72.00)	47.25 ± 12.66
**NRS 1st month**	4.00 (0.00–8.00)	3.56 ± 1.70	ODI 1st month	22.00 (4.00–68.00)	26.61 ± 17.51
**NRS 3rd month**	3.00 (0.00–8.00)	3.20 ± 1.95	ODI 3rd month	20.00 (2.00–70.00)	24.88 ± 18.44
**NRS 6th month**	3.00 (0.00–8.00)	3.24 ± 2.03	ODI 6th month	20.00 (2.00–70.00)	24.54 ± 18.79

NRS: Numerical Rating Scale, ODI: Oswestry Disability Index; min–max; minimum–maximum; SD: standard deviation.

**Table 5:** The differences between the averages of NRS and ODI variables obtained from patients before treatment, at 1 month, 3 months, and 6 months, and the significance test results of these differences.

	NRS Pre-Procedural	NRS 1st Month	NRS 3rd Month	NRS 6th Month	ODI Pre-Procedural	ODI 1st Month	ODI 3rd Month	ODI 6th Month
**NRS Pre-Procedural**		3.864 *p* = 0.000	4.220 *p* = 0.000	4.186 *p* = 0.000				
**NRS 1st Month**			0.356 *p* = 0.002	0.322 *p* = 0.007				
**NRS 3rd Month**				−0.034 *p* = 0.705				
**NRS 6th Month**								
**ODI Pre-Procedural**						20.644 *p* = 0.000	22.373 *p* = 0.000	22.712 *p* = 0.000
**ODI 1st Month**							1.729 *p* = 0.002	2.068 *p* = 0.001
**ODI 3rd Month**								0.339 *p* = 0.154
**ODI 6th Month**								

NRS: Numerical Rating Scale, ODI: Oswestry Disability Index, *p* < 0.05.

## Data Availability

Research data supporting this publication were obtained from the patient history database.
